# The Influence of Micronutrients in Cell Culture: A Reflection on Viability and Genomic Stability

**DOI:** 10.1155/2013/597282

**Published:** 2013-05-27

**Authors:** Ana Lúcia Vargas Arigony, Iuri Marques de Oliveira, Miriana Machado, Diana Lilian Bordin, Lothar Bergter, Daniel Prá, João Antonio Pêgas Henriques

**Affiliations:** ^1^Laboratório de Reparação de DNA em Eucariotos, Departamento de Biofísica/Centro de Biotecnologia, UFRGS, Avenida Bento Gonçalves 9500, Prédio 43422, Setor IV, Campus do Vale, 91501-970 Porto Alegre, RS, Brazil; ^2^Instituto de Educação para Pesquisa, Desenvolvimento e Inovação Tecnológica—ROYAL, Unidade GENOTOX—ROYAL, Centro de Biotecnologia, UFRGS, Avenida Bento Gonçalves 9500, Prédio 43421, Setor IV, Campus do Vale, 91501-970 Porto Alegre, RS, Brazil; ^3^PPG em Promoção da Saúde, Universidade de Santa Cruz do Sul (UNISC), Avenida Independência 2293, 96815-900 Santa Cruz do Sul, RS, Brazil; ^4^Instituto de Biotecnologia, Departamento de Ciências Biomédicas, Universidade de Caxias do Sul (UCS), Rua Francisco Getúlio Vargas 1130, 95070-560 Caxias do Sul, RS, Brazil

## Abstract

Micronutrients, including minerals and vitamins, are indispensable to DNA metabolic pathways and thus are as important for life as macronutrients. Without the proper nutrients, genomic instability compromises homeostasis, leading to chronic diseases and certain types of cancer. Cell-culture media try to mimic the *in vivo* environment, providing *in vitro* models used to infer cells' responses to different stimuli. This review summarizes and discusses studies of cell-culture supplementation with micronutrients that can increase cell viability and genomic stability, with a particular focus on previous *in vitro* experiments. In these studies, the cell-culture media include certain vitamins and minerals at concentrations not equal to the physiological levels. In many common culture media, the sole source of micronutrients is fetal bovine serum (FBS), which contributes to only 5–10% of the media composition. Minimal attention has been dedicated to FBS composition, micronutrients in cell cultures as a whole, or the influence of micronutrients on the viability and genetics of cultured cells. Further studies better evaluating micronutrients' roles at a molecular level and influence on the genomic stability of cells are still needed.

## 1. Introduction

Micronutrients, essential nutrients that are needed in small amounts, are as important for life as macronutrients. Micronutrients comprise all of the vitamins, such as A, D, and E, as well as the minerals, such as calcium, zinc, and iron. The *in vivo *role of micronutrients is well established, and several studies have examined the effects of micronutrients on genomic stability [1–21]. Approximately 40 micronutrients are required in the human diet, and for each micronutrient, proper metabolism demands an optimal level of intake. A micronutrient deficiency distorts the metabolism in numerous and complicated ways, many of which may lead to DNA damage.

Micronutrients are required for optimal macronutrient metabolism because of micronutrients' critical role in intermediate metabolism. Invariably, metabolism requires the concomitant involvement of one or more vitamins and minerals. Chronic degenerative disease etiology and the rate of pathogenesis are thus intimately associated with micronutrient imbalances. Nutrition research has recently highlighted the role of several nutrients in regulating the genomic machinery [22]. More specifically, a number of vitamins and micronutrients are substrates and/or cofactors in the metabolic pathways regulating DNA synthesis and/or repair and gene expression [23]. A deficiency in such nutrients may result in the disruption of genomic integrity and alteration of DNA methylation, thus linking nutrition with the modulation of gene expression. In many cases, the response to a nutrient deficiency also seems to be genotype specific. Gene-nutrient interactions are thus a fascinating example of physiological responses to the environment/diet at the molecular level [22].

Minerals and vitamins are indispensable to DNA metabolic pathways [24, 25]. Although there is still no clear evidence for a diet that optimally protects against DNA damage, in terms of either proportions or combinations of specific micronutrients, many studies that conducted *in vitro* and in animal models have demonstrated the roles of micronutrients in maintaining genomic stability. For example, vitamins C and E deficiencies are known to cause DNA oxidation and chromosomal damage [26, 27]. Vitamin D exhibits antioxidant activity, stabilizes chromosomal structure, and prevents DNA double-strand breaks [28]. Similarly, magnesium is an essential cofactor in DNA metabolism that plays a role in maintaining the high fidelity of DNA transcription [29]. Whereas either an excess of or a deficiency in iron may cause DNA breaks [30], a carotenoid-rich diet reduces DNA damage [31], but excess retinol may be carcinogenic in certain individuals [32]. In a final example, vitamin B-12 deficiency is associated with the formation of micronuclei [5, 24], and reduced transcobalamin II in the serum is associated with chromosomal abnormalities [33].

Given the importance of micronutrients *in vitro*, the optimization of cell viability and genomic stability warrants further studies. Cell-culture media mimicking the *in vivo* environment may help to generate *in vitro* models of a cell's response to different stimuli. The composition of these media includes certain vitamins and minerals, but unfortunately, in many common culture media, the only source of micronutrients is fetal bovine serum (FBS), which contributes to only 5–10% of the media composition. Moreover, the appropriate proportion of micronutrients is not always provided because the precise composition of each batch of FBS is in fact extremely variable [34].

Certain micronutrients, such as calcium, folate, magnesium, and iron, have been reported as key elements in cellular processes, including the proliferation, survival, and even differentiation of cell cultures [35–38]. However, the particular concentration of micronutrients in a culture as well as the cell type may trigger different responses. Further studies of micronutrients' roles at a molecular level and influence on genomic stability are still required.

## 2. Aims and Scope

This review summarizes and discusses studies showing the influence of some micronutrients on cell viability and genomic stability, with a particular focus on *in vitro* models. *In vivo* evidences are presented to illustrate the relevance of the nutrients to genomic stability. Papers were retrieved from PubMed using the following search terms: micronutrients, vitamins, minerals, cell culture, proliferation, viability, and genomic stability. Additional publications were collected by cross-referencing the primary articles retrieved. The review does not aim to include all nutrients that could influence genomic stability; then, only the following nutrients were included vitamins A, B7, B9, B12, C, and E and minerals Cu, Fe, Mg, Se, and Zn. According to Friso and Choi [39], an imbalance of such dietary nutrients as folate, zinc, vitamin C, and selenium can alter genomic and/or gene-specific DNA methylation, resulting in many different molecular effects on gene expression and integrity, in turn affecting cell growth, tissue differentiation, cancer incidence, and aging. To better address the selected micronutrients' effects in cell viability and genomic stability, we considered the information available regarding either their deficiency or excess.

## 3. Micronutrients and Their Influence on Genomic Stability

DNA damage is one of the most important factors that can compromise homeostasis, resulting in chronic (e.g., atherosclerosis) and even degenerative diseases, including Alzheimer's disease (AD) and certain types of cancer [40]. A deficiency in or imbalance of certain micronutrients has been described as mimicking radiation or chemicals, causing single- and double-strand breaks (SB) or lesions in DNA, or even both [20].

In Table 1, micronutrients whose imbalances cause DNA damage are listed, as well as the nutrients' food sources and possible health effects. In general, micronutrients can either act directly on the genome to prevent mutations or protect the genome indirectly by serving as enzyme cofactors in the cellular processes that modulate transformation [41, 42]. Therefore, any imbalance may result in a degree of DNA damage.

The role of diet in determining genomic stability is more important than previously imagined. It has been found that diet affects all pathways relevant to genomic stability, including exposure to dietary carcinogens, activation and detoxification of carcinogens, DNA repair, DNA synthesis, and cell apoptosis [23, 43]. All of these critical pathways are dependent not only on enzymes but also on substrates and cofactors, a few of which are only available at the right concentration when the dietary intake of key minerals and vitamins is adequate [44]. As a result, a dietary deficiency in certain micronutrients required for DNA maintenance may exert effects similar to inherited genetic disorders that impair the activity of enzymes required for genomic stability [23, 45–47]. Additionally, such a deficiency may damage DNA to a similar extent as significant exposure to known carcinogens, such as ionizing radiation [43].

### 3.1. Vitamin A

Vitamin A is also referred to as retinoic acid, retinol, retinal, *α*- and *β*-carotene, lycopene, lutein, zeaxanthin, *β*-cryptoxanthin, or astaxanthin. The role of vitamin A and provitamin A (carotenoids) in DNA damage has recently been reviewed by Azqueta and Collins [65]. The well-established antioxidant properties of vitamin A have facilitated studies measuring oxidative damage both *in vivo*, in animal studies and human clinical trials, and *in vitro*. Whereas high concentrations of provitamin A carotenoids can cause DNA damage, perhaps by acting as prooxidants, nonvitamin A carotenoids that can significantly reduce such damage [66].

The functions of vitamin A are related to night, day, and color vision; epithelial-cell integrity against infections; the immune response; hemopoiesis; skeletal growth; male and female fertility; embryogenesis. Paradoxically, either an excess of or a deficiency in retinoic acid results in similar malformations in certain organs, including the mammalian kidney [67]. Many eye pathologies are due to vitamin A deficiency, including night blindness, conjunctival xerosis and corneal injuries. Similarly, hypervitaminosis A, resulting from the storage of excess vitamin A in the body, can damage various systems. Very large doses of vitamin A, especially in young children, can increase the intracranial pressure, leading to headache, nausea, and vomiting [68]. It has also been established that adequate vitamin A intake is required for normal organogenesis, immune function, tissue differentiation, and vision. Given these requirements, vitamin A deficiency, which is widespread in the developing world, is responsible for at least one million instances of unnecessary death and blindness each year [69].

### 3.2. Vitamin B7

Vitamin B7, also known as biotin, acts as a cofactor for the biotin-dependent enzymes pyruvate carboxylase, propionyl-CoA carboxylase, crotonyl-CoA carboxylase, and two isozymes of acetyl-CoA carboxylase [70]. These enzymes catalyze key steps in important metabolic pathways, including fatty acid biosynthesis, gluconeogenesis, and amino acid metabolism [71]. Vitamin B7 deficiency due to inadequate dietary intake or congenital defects in biotin absorption or metabolism results in the inactivation of all five biotin-dependent enzymes. This condition is known as multiple carboxylase deficiency (MCD) [72, 73], whose symptoms include ketoacidosis, lactic acidosis, feeding difficulties, skin rashes, and neurological abnormalities, such as subependymal cysts, hypotonia, seizures, and ataxia. In severe cases, or if MCD is left untreated, the condition can lead to coma or death [74].

It has been demonstrated that biotin plays a role in DNA-strand breaks and the cellular response to strand breaks (SB). More specifically, biotin supplementation increased DNA breaks in cell cultures, although it is unknown whether this finding is relevant to whole organisms [75]. In contrast, *in vivo*, a high biotin intake in combination with a low intake of multiple other nutrients has been associated with increased genomic stability [53]. Biotin deficiency rarely occurs spontaneously in animals, including humans [76], but can be induced by consuming large amounts of raw egg white, which contains avidin, known to inhibit biotin absorption from the intestinal tract, or by taking anticonvulsants [77].

### 3.3. Vitamin B9

A deficiency in vitamin B9, also known as folic acid or folate, is common in people who consume few fruits and vegetables. Vitamin B9, as well as other vitamins from the B complex, plays an important role in genomic stability, and a deficiency can cause chromosomal breaks in human genes [78]. Vitamin B9 deficiency can also lead to (a) an elevated rate of DNA damage and altered DNA methylation, both of which are risk factors for cancer [78–80], possibly including colon cancer [81] or (b) an increased homocysteine concentration, an important risk factor for cardiovascular disease [82]. These defects may also play a significant role in developmental and neurological abnormalities [78, 79]. However, in animals with existing preneoplastic or neoplastic lesions, folicacid supplementation increases the tumor burden [83]. In contrast, the adequate intake of vitamin B9 can increase genomic stability and possibly reduce cancer risk [84–87] because vitamin B9 is a key carbon donor during nucleotide biosynthesis [88].

### 3.4. Vitamin B12

Vitamin B12, or cyanocobalamin, deficiency is associated with pernicious anemia and neurological pathologies varying from a minor decrease in cognitive function to neurodegenerative disorders, although the role of vitamin B12 in these conditions requires further investigation [89, 90]. The lack of understanding of the underlying molecular mechanisms may be due to the experimental limitations of the available classical cell-culture models [89]. Nevertheless, vitamin B12 is known to play an important role in genomic stability, and a deficiency in vitamin B12 can lead to DNA damage [81]. Vitamin B12 is also required for the synthesis of methionine and *S*-adenosyl methionine, the common methyl donor required for the maintenance of the DNA methylation patterns that determine gene expression and DNA conformation [91].

Despite controversies in the literature regarding the prevalence of vitamin B12 deficiency, this deficiency seems to be more common among people aged 65–76 years [92]. However, the symptoms of vitamin B12 deficiency caused by poor diet, digestive problems, and/or inadequate absorption in elderly people can be nonspecific, rendering a diagnosis more difficult. Furthermore, neurological symptoms may appear before anemia; in fact, only approximately 60% of elderly people with vitamin B12 deficiency are anemic [92, 93]. In cell-culture models, sufficient vitamin B12 can be provided to the cells by the FBS [89].

### 3.5. Vitamin C

Vitamin C, also known as ascorbate or ascorbic acid, is a micronutrient required for innumerable biological functions, specifically serving as a cofactor for certain important enzymes [94]. One type enzyme is the prolyl hydroxylases, which play a role in collagen biosynthesis and the downregulation of hypoxia-inducible factor- (HIF-) 1, a transcription factor that regulates many genes responsible for tumor growth, energy metabolism, and neutrophil function and apoptosis. Vitamin C-dependent inhibition of the HIF pathway may provide alternative or additional approaches to controlling tumor progression, infection, and inflammation [94].

As vitamin C exhibits antioxidant properties that provide protection against oxidative stress-induced cell damage by scavenging reactive oxygen species (ROS), the effects of this vitamin on cancer chemoprevention [95, 96] and cancer treatment [97] as well as sepsis [98] and neurodegenerative diseases (e.g., Alzheimer's disease) [99] have been studied. In fact, ingesting inadequate levels of vitamin C can mimic radiation exposure. In the literature, numerous human supplementation studies have used biomarkers of oxidative damage to DNA, lipids (lipid oxidation releases mutagenic aldehydes), and protein. Although these studies have yielded both positive and negative results, if the fact that blood-cell saturation occurs at approximately 100 mg/day is taken into consideration, the evidence suggests that this level of vitamin C intake minimizes DNA damage [20]. Unfortunately, vitamin C deficiency is common in poor communities, so measures to improve the consumption of vitamin C-rich foods should be considered [100].

### 3.6. Vitamin E

Vitamin E, which comprises compounds from the tocopherol and tocotrienol families, is required to prevent peripheral neuropathy and hemolytic anemia in humans, which arise due to vitamin E deficiency. Vitamin E functions as a vital lipid-soluble antioxidant, scavenging hydroperoxyl radicals in the lipid milieu. The human symptoms of vitamin E deficiency suggest that this vitamin's antioxidant properties play a major role in protecting erythrocyte membranes and nervous tissues [94]. Additionally, these antioxidant properties play a role in genomic stability, particularly because vitamin E is a potent peroxyl radical scavenger. Vitamin E is also a chain-breaking antioxidant that prevents the propagation of free radicals in membranes and plasma lipoproteins [101].

Recently, Ni and Eng [102] demonstrated that *α*-tocopherol can selectively protect SDH (var^+^) cells from oxidative damage and apoptosis and rebalance the redox metabolites nicotinamide adenine dinucleotide (NAD^+^ and NADH). Another interesting recent study [103] evaluated the amount of the oxidation product 8-oxo-7,8-dihydro-2′-deoxyguanosine (8-oxodG) formed from the DNA nucleoside deoxyguanosine (dG) after vitamin exposure. In the case of vitamin E, no DNA damage was induced in cultured cells. Taken together, these results reinforce the role of this vitamin in maintaining DNA integrity and stability. Although the direct comparison of the study outcomes is complicated by varying definitions of vitamin E deficiency, the available data suggest that children and the elderly are most vulnerable to this deficiency and that men may be at higher risk than women [104].

### 3.7. Copper

Copper is an essential trace element, serving as a cofactor for many enzymes in different biological processes. In contrast to iron, the copper concentration not only in the blood but also in individual organs is maintained at constant levels beginning in early childhood, indicating the presence of robust homeostatic mechanisms [105]. Adequate copper intake permits the normal utilization of dietary iron, as intestinal iron absorption, iron release from stores (e.g., in the macrophages of the liver and spleen), and iron incorporation into hemoglobin are copper-dependent processes. In addition to preventing anemia, copper assists in blood coagulation and blood-pressure control; the crosslinking of connective tissue in the arteries, bones, and heart; defense against oxidative damage; energy transformation; the myelination of the brain and spinal cord; reproduction; hormone synthesis. In contrast, inadequate copper intake has adverse effects on the metabolism of cholesterol and glucose, blood pressure control and heart function, bone mineralization, and immunity [106].

The excessive accumulation of copper in the body can contribute to the development of cancer due to copper's role in causing DNA damage [107]. Curiously, in addition to the robust mechanisms maintaining copper homeostasis and copper's rapid excretion, mammals express copper-dependent enzymes that are central players in antioxidant defense. Thus, whereas copper can induce ROS formation when involved in Fenton-like or Haber-Weiss reactions, copper-dependent processes can also help to clear ROS [105]. For further information on the relationship between copper and DNA damage, please refer to the recent review published by Linder [105].

### 3.8. Iron

Iron is a crucial nutritional element for all life forms that plays a critical role in the cell, including electron transport and cellular respiration, proliferation and differentiation, and the regulation of gene expression [3]. Iron can undergo univalent redox reactions, resulting in oxidized and reduced forms known as ferric (Fe^3+^) and ferrous (Fe^2+^) iron, respectively. Due to iron's oxireduction, which can contribute to ROS generation, as well as iron's role in Fenton and Haber-Weiss reactions, this nutrient is also potentially deleterious. These reactions occur when an inorganic nutrient, such as Fe^2+^ or Cu^+^, is in excess and donates an electron to H_2_O_2_, leading to OH production. The ROS generated by Fenton chemistry can contribute to major pathologies, such as cancer, atherosclerosis, and neurodegenerative diseases [38].

Free radicals can cause serious damage to the genome. Depending on the dose and type, inorganic nutrients can protect against or contribute to oxidative stress [108]. Peroxidases and especially catalase, which use heme iron as a cofactor, decompose H_2_O_2_. If the resultant reactive species are not efficiently removed, these species can induce the formation of the more active OH or peroxynitrite, which may result in DNA oxidation. Therefore, deficiencies in such nutrient-dependent antioxidant enzymes can increase oxidative stress and favor the genomic instability [109].

In addition, iron is a cofactor of many important enzymes related to DNA repair mainly as clusters of iron sulphur. For example, the glycosylases MutyH and NTHL1 involved in base excision repair (BER) and mismatch repair (MMR) and the helicases ERCC2 and BACH1 acting in the nucleotide excision repair (NER) possess iron-sulphur clusters in their structure [110, 111]. The increased DNA damage sensitivity in cells with impaired Fe/S protein biogenesis may include the loss of nucleotide excision repair because maturation of XPD is defective. Since the Fe/S cluster of XPD is required for its DNA helicase activity *in vitro* [110].

Although excess iron can cause oxidative DNA damage in rats and has been associated with an increased risk of cancer and heart disease in humans [20], iron deficiency also appears to lead to oxidative DNA damage and is associated with cognitive dysfunction in children. The importance of iron in normal neurological function has been well established, as neurons require iron for many physiological processes, including electron transport and axonal myelination, and as a cofactor for many enzymes involved in neurotransmitter synthesis [112, 113]. In contrast, inadequate iron intake results in anemia, immune dysfunction, and adverse pregnancy outcomes, such as premature birth. Maintaining physiological iron levels via dietary intake is thus mandatory for health. However, iron deficiency is still very common in the human population, particularly among children and pregnant women [114].

### 3.9. Magnesium

Magnesium is indispensable to life, as this micronutrient is involved in many important biological processes. Magnesium has multiple functions in all cellular processes, including DNA replication and protein synthesis, and also serves as a cofactor for DNA-repair proteins and in the maintenance of a cell's redox status, cell-cycle regulation, and apoptosis [29]. Magnesium deficiency or the displacement of Mg^2+^ by other toxic, divalent metal ions leads to increased genomic instability, which has been implicated in many diseases [115] and may result in inhibited DNA repair, oxidative stress, accelerated aging, and increased cancer risk [29, 116]. Studies have indicated that higher magnesium consumption may protect against certain inflammatory disorders, such as insulin resistance [117], hypertension [118], diabetes mellitus [119], and cardiovascular disease [118].

Magnesium is not genotoxic at physiologically relevant concentrations and in fact maintains low mutation frequencies by facilitating high-fidelity replication and by supporting all DNA-repair processes and chromosomal segregation during mitosis [29]. In fact, it is an essential cofactor in NER, BER, and MMR processes, where magnesium is required for the removal of DNA damage [120]. All downstream activities of major base excision repair proteins, such as apurinic/apyrimidinic endonuclease, DNA polymerase beta, and ligases, require magnesium. Thus, this element may act as a regulator for the base excision repair pathway for efficient and balanced repair of damaged bases, which are often less toxic and/or mutagenic than their subsequent repair product intermediates [121]. Magnesium is also important for the fidelity of DNA replication, impacting cell cycle and apoptosis [61].

Animal and human epidemiological studies have demonstrated inverse correlations between magnesium levels and cardiovascular disease [29] or the incidence of certain types of cancer, including colorectal cancer [122, 123]. Additionally, magnesium deficiency is one risk factor for premature aging [29]. The relationship between magnesium levels and tumorigenesis is more complex, with magnesium deficiency increasing tumor incidence in animals and humans, whereas magnesium promotes the growth of preexisting tumors due to profound changes in magnesium homeostasis in tumor cells. Thus, the protective effects of magnesium are restricted to the early stages of tumor development [29]. According to Ford and Mokdad [124], despite the role of magnesium in maintaining good health, historically, much of the population of the United States has not consumed adequate amounts of this nutrient. Additionally, there are significant racial and ethnic disparities in magnesium intake.

### 3.10. Selenium

The trace element selenium is another well-established micronutrient essential for mammalian health [125]. Selenium is a constituent of the small group of selenocysteine-containing selenoproteins [126], including glutathione peroxidase, thioredoxin reductase, selenoprotein P, and selenoprotein R, which are primarily involved in antioxidant activity and the maintenance of a cell's redox state [127–130]. Due to selenium's key role in redox regulation and antioxidant function, this nutrient is critical for membrane integrity, energy metabolism, and protection against DNA damage [126]. However, in certain cases, selenium can also lead to oxidative DNA damage [20], increased infection risk, and altered mood [131]. Whether selenium exerts positive or negative effects *in vivo* or *in vitro* is related to dose. Interest in organoselenide chemistry and biochemistry has increased over the last two decades, mainly because a variety of organoselenium compounds can be used as antioxidants, enzyme inhibitors, neuroprotective, antitumoral, or anti-infectious agents, as well as cytokine inducers and immunomodulators [125, 132–135]. In fact, an interaction with the zinc finger structures of DNA repair proteins may occur by essential trace elements such as certain selenium compounds, which appear to exert anticarcinogenic properties at low concentrations but may compromise genetic stability at higher concentrations [136].

Selenium deficiency alone is not common in developed countries, but an inadequate intake of this mineral has been associated with the development of cancer, asthma, and coronary disease, among other chronic conditions [137]. When required, dietary supplementation must be performed carefully, given the intrinsic toxicity of high selenium levels [138].

### 3.11. Zinc

Zinc is one of the most important micronutrients due to the prevalence of zinc-dependent enzymes in metabolic processes; zinc's vital role in several bodily functions, such as vision, taste perception, cognition, cell reproduction, growth, and immunity; the beneficial effect of zinc supplementation on many disease states [139]. In fact, zinc is a component of over 300 proteins, including over 100 DNA-binding proteins with zinc fingers, Cu/Zn superoxide dismutase, the estrogen receptor, and the synaptic transmission protein [20]. Zinc also has a crucial role in the biology of p53, in that p53 binds to DNA through a structurally complex domain stabilized by zinc atom, possibly increasing the response to anticancer drugs [140].

Zinc deficiency is a health problem in many communities, especially among adolescents, due to the pubertal growth spurt [139]. At the molecular level, there is evidence of a relationship between zinc deficiency and increased chromosomal breaks, possibly due to increased oxidative damage stemming from a loss in the activity of Cu/Zn superoxide dismutase or the zinc-containing DNA-repair enzyme Fapy glycosylase, which repairs oxidized guanine [20]. Unfortunately, nearly half of the world's population is at risk of inadequate zinc intake, so public health programs are urgently needed to reduce zinc deficiency [139].

### 3.12. Summary of the Effects of the Selected Micronutrients on Genomic Stability

Taking the preceding discussion and other evidence from the literature into account, the adequate intake of micronutrients seems to have an important role in genomic stability. In contrast, an imbalance of the same micronutrients may also negatively impact the DNA, possibly via oxidative stress, consequently causing or contributing to different human diseases. It is thus highly relevant to elucidate the mechanism underlying the response to and repair of oxidative stress and this mechanism's relationship to the DNA damage response pathways, all of the inorganic nutrients (vitamins and minerals) and disease, including carcinogenesis. An understanding of the possible influences on genomic stability, even in cell culture, is also in current demand.

## 4. Cell-Culture Medium and Micronutrients That Increase Genomic Stability: Is the Concentration Relevant?

According to Ferguson and Fenech [141], the last decade of studies on micronutrients and genomic stability have improved dietary recommendations based on the prevention of DNA damage or the maintenance of genomic integrity. In light of this, the development of *in vivo* and especially *in vitro* models to more robustly evaluate DNA damage is necessary.

Table 2 presents interesting data regarding the micronutrients that may interfere with genomic stability and the micronutrient concentration values found in typical cell-culture media, FBS, and human serum. Unfortunately, data are not available for all of the micronutrients in the media, and even the proportions of micronutrients in FBS, as an organic product, are not all well characterized. Additionally, as demonstrated by Bryan et al. [34], the concentration of many micronutrients in FBS can vary significantly between batches.

Although cell-culture media attempt to provide an environment similar to the *in vivo* milieu of cell development, there is an evident imbalance of micronutrients between the media and human serum. Certain micronutrients are present in these media at concentrations higher than those found in human serum (e.g., vitamins B7 and B12), whereas other nutrients are present at significantly lower concentrations than in human serum (e.g., iron and zinc). A recent study [103] called attention to the composition of multivitamin supplements, which may trigger unwanted health outcomes due to the synergistic oxidative effects of the component vitamins and metals. In this research, the vitamins' chemical oxidation potencies were studied by measuring the amount of the oxidation product 8-oxo-7,8-dihydro-2′-deoxyguanosine (8-oxodG) formed from the DNA nucleoside deoxyguanosine (dG) after vitamin exposure. The micronutrients evaluated by the authors were the vitamins A, B1, B2, B3, B6, B12, and C; *β*-carotene; folic acid; *α*-, *δ*-, and *γ*-tocopherol. The minerals copper, iron and zinc were also examined. All of these micronutrients were tested in cell culture, alone or in combination, taking the human serum levels of each micronutrient into account. The main conclusion reported was that certain vitamins, alone or in combination with metals (e.g., vitamin C and copper), can induce DNA damage. However, cells in culture and *in vivo* have distinct needs for nutrients and growth factors, as the cells' activity in each environment may differ due to interactions with other cells or parts of the larger organism. Thus, examining physiological concentrations of micronutrients *in vitro* may not be the most appropriate approach.

As mentioned above, each cell type may have a distinct requirement for micronutrients. Depending of the origin of the cell and its role *in vivo*, the cell may specifically have a higher affinity for one micronutrient over another. In the case of iron, for example, which is stored in specific tissues, including the spleen, liver, and bone marrow [142], the primary cells or immortal cell lines derived from these tissues may have a greater need for this specific micronutrient. In the case of certain neuronal cells, which require iron for cell development [143], the demand for iron may also be higher than in other cell types. Although the evaluation of micronutrients' influence on DNA damage and integrity as well as on cell development, including the related enzymes and proteins, should be continued, the micronutrient concentrations relevant not only to human but also to cell-culture genomic stability must be considered.

## 5. Could Changes in a Culture's Micronutrient Composition Influence the Viability and Genetics of the Cultured Cells?

Cells are typically maintained at an appropriate temperature and CO_2_ concentration (usually 37°C and 5% CO_2_ for mammalian cells) in an incubator. Beyond these parameters, the most commonly varied factor in culture systems is the growth medium. The recipes for growth medium can vary in pH, glucose concentration, growth factors and the presence of other nutrients and micronutrients. The development of synthetic basal formulations for mammalian cell-culture applications has been facilitated by the contributions of many investigators. In particular, the definition of the minimally required nutrients by Harry Eagle in the 1950s spawned an iterative process of continuous modification and refinement of the exogenous environment to cultivate new cell types and support the emerging applications of cultured mammalian cells. This process led to the development of highly potent, basal nutrient formulations capable of sustaining serum-free cell proliferation and biological production [152]. However, the growth factors most often used to supplement cell-culture media are still derived from animal blood, such as FBS. FBS has become the supplement of choice for cell culture-based research, containing an array of proteins, growth factors, and ions necessary for cell viability and proliferation *in vitro*, including certain vitamins and minerals [153]. Currently, the use of these ingredients is minimized or eliminated wherever possible in favor of chemically defined media, but this substitution is not always possible.

Bryan et al. [34] stated that one of the major obstacles to obtaining human cells of a defined and reproducible standard, and thus suitable for use in medical therapies, is the routine necessity of supplementing cell-culture media with FBS. In this study, FBS variants were evaluated, in terms of both elemental (micronutrient) composition and the variants' effects on the expression of a group of proteins associated with the antigenicity of primary human umbilical vein endothelial cells (HUVECs). A combination of inductively coupled plasma mass spectrometry (ICPMS) and flow cytometry was used to achieve these experimental objectives. Statistically significant differences in antigenic expression during cell culture were demonstrated for a set of trace elements in FBS (e.g., lithium, boron, magnesium, phosphorus, sulfur, potassium, titanium, vanadium, chromium, manganese, iron, copper, zinc, gallium, and selenium). The lack of reproducibility and the variation in protein expression in the primary human cells was attributed to the FBS supplementation.

Culture conditions for cell lines are known to affect gene expression [154–156], while stem cells grown in different types of serum exhibit variable differentiation and proliferation characteristics [157, 158] the same cell line, if cultivated in different conditions, can present different phenotypes. Nevertheless, the cellular requirement for a specific micronutrient is directly correlated with the cell type, the rate of cell grow, and the stage of cell differentiation. In light of this, it is important to observe that minimal attention has been dedicated to the composition of FBS and the micronutrient supplementation of media in cell cultures or the fact that micronutrients can influence the viability and genomic stability of cultured cells.

In Tables 3 and 4, a few examples of the effects of vitamins and minerals in cell culture and on genomic stability, drawn from the literature, are highlighted.

### 5.1. Vitamin A

For vitamin A, but possibly applicable to many other micronutrients, the studies presented in Table 3, conducted at low concentrations, which tend to show protective effects, whereas higher concentrations are associated with increased DNA damage [65]. This finding is consistent with the known ability of *β*-carotene to act as a prooxidant, rather than as an antioxidant, at high concentrations and under high oxygen tension [178]. The physiological concentrations of micronutrients should always be evaluated and, if possible, at least used as a maximum in studies evaluating the viability and genomic stability of cell cultures. However, as can be verified in Table 2, there is a lack of data regarding the presence of vitamin A in cell-culture media.

### 5.2. Vitamin B7 (Biotin)

Biotin plays an important role in regulating gene expression, thus mediating certain aspects of cell biology and fetal development [179]. The effects of biotin deficiency are detailed in Table 3 and are related to decreased rates of cell proliferation, impaired immune function, and abnormal fetal development. An excess of biotin is also mentioned and can exert reproductive and teratogenic effects. However, as can be verified in Table 2, cell-culture media containing higher levels of biotin than human serum are common. More studies evaluating the effects of the high biotin levels in cell cultures are necessary.

### 5.3. Vitamin B9

Folate depletion appears to enhance carcinogenesis, whereas folate supplementation above what is presently considered to be the basal requirement confers a protective effect [180]. A few examples of folate deficiency and supplementation are described in Table 3, and the relationship between this vitamin and cell proliferation and apoptosis has been demonstrated. Furthermore, as can be verified in Table 2, the folate levels in the cell-culture media evaluated are typically higher than those levels found in human serum. It is well established that folate deficiency can influence the genomic stability of cultured cells [81, 181], yet there is still a lack of data evaluating whether folate levels above the physiological range can impair cell growth. Elevated levels of folic acid should be examined, as in tumor-prone animals, both folate deficiency and supplementation promote the progression of established neoplasms [83, 182]. As a folate overload is more common than a deficiency in *in vitro* studies, the former should be most thoroughly evaluated.

### 5.4. Vitamin B12

Vitamin B12 deficiency has been described as similar to chemicals that damage DNA by causing single- and double-strand breaks [20]. As demonstrated in Table 3, in a cellular model designed to better understand vitamin B12 deficiency in the brain, the growth and differentiation of neuronal cells were affected [89]. Additionally, supplementation with certain cobalamin compounds protected the cells from neurotoxicity and increased cell growth [170, 171]. Unfortunately, *in vitro *research demonstrating a direct link between vitamin B12 deficiency or overload and genomic stability in human cells has not yet been published. Based on Table 2, however, high concentrations of vitamin B12 are more common in cell-culture media than in human serum.

### 5.5. Vitamin C

In Table 3, a few examples of the influence of vitamin C in cell cultures are provided. Different concentrations of this vitamin result in distinct responses, ranging from DNA damage (at higher concentrations) to the protection of DNA (at lower concentrations). Importantly, the concentration of vitamin C in current cell cultures is not available in Table 2, as possibly only trace levels are present in media. As the cellular response to vitamin C may be dose-dependent, a similar concentration of this vitamin in culture media to that in human serum should be evaluated.

### 5.6. Vitamin E

*In vivo* vitamin E supplementation is still being discussed [183], and more *in vitro* studies will be required to better understand the protective effects of vitamin E on cell viability and genomic stability. Nevertheless, certain results (Table 3) have been consistent with the concept that *α*-tocopherol, combined with ascorbic acid or alone, can protect against oxidative DNA damage [175] and reduce apoptosis and autophagy [177] under certain conditions. Unfortunately, the current *in vitro* concentration of vitamin E is also not available in Table 2, as possibly only trace levels are present in media. Given this observation, it is interesting to observe that the *in vitro* studies of vitamin E described in Table 3 adopted concentration values similar to that of human serum (approximately 30 *μ*mol/L) and that the results were positive for the cell cultures.

### 5.7. Copper

As can be verified in Table 2, there is a marked lack of copper in common cell-culture media, even when supplemented with FBS. Thus, cells in culture are typically exposed to an environment deficient in a micronutrient critical for the formation of detoxifying enzymes, which may impact cell development and possibly genomic stability and survival rates. It is important to note that the copper concentrations evaluated in cell culture (Table 4) are generally above the human physiological range, so toxic effects in cultures should be expected. Thus, the optimization of the copper concentration in cell cultures is necessary to maintain cell viability and genomic stability and to avoid the deleterious effects of this metal.

### 5.8. Iron

In Table 4, it is important to note that the results of Lima et al. [187] may be expected in a cell culture in which the requirements for micronutrients are quite different from those *in vivo*. In this study, the concentrations evaluated were generally higher than the values measured in human serum (Table 2), and even the lowest concentration of iron applied for the authors (22.38 *μ*mol/L) would be considered high for cells in culture. For HL-60 leukemia cells, as demonstrated in [201], the iron concentration range for optimal cell proliferation is very narrow (2-3 *μ*mol/L). In contrast, in the studies in which the iron levels were between 5 and 10 *μ*mol/L, these levels generally benefitted the cultures analyzed, or at least no damage was observed [187–189].

### 5.9. Magnesium

As presented in Table 4, several studies on the effects of magnesium deficiency on cultured cells have demonstrated reduced oxidative stress, cell-cycle progression, cell growth, and cell viability [190, 191, 202–207]. Killilea and Ames [192] specifically investigated the consequences of long-term and moderate magnesium deficiency in normal human cells in comparison with more typical magnesium levels, using a concentration observed in normal human serum (0.8 mmol/L). No alterations were observed in the cells cultured in the medium containing normal magnesium levels. Additionally, based on studies conducted either in bacteria or in mammalian cells in culture, there is no evidence for the genotoxic effects of magnesium salts at physiologically relevant doses [29], indicating that adequate micronutrient levels in cell-culture media may improve cell viability and genomic stability. As shown in Table 2, the levels of magnesium currently found in cell-culture media are very similar to those levels in human serum, which is very unusual for micronutrients in general.

### 5.10. Selenium

The differential toxicities elicited by selenocompounds need to be taken into account in *in vivo* and *in vitro* supplementation studies [194]. The references in Table 4 evaluated different forms of selenium and certain salts that may be more toxic to the cellular environment than others. Due to the importance of selenium as well as many other micronutrients discussed in this review, the micronutrient concentration in the media, as well as the FBS, intended for cell culture should be controlled and adjusted to the physiological range, if applicable. By comparing the human serum concentration of selenium in Table 2 with those concentrations described in the experiments cited in Table 4, it is apparent that the concentrations below the physiological range benefitted the cell culture, although high concentrations of selenium compounds potentially negatively affected tumor cells.

### 5.11. Zinc

The role of zinc in genomic stability was recently reviewed by Sharif et al. [208]. Additionally, a few brief examples of zinc's influence on cell viability and genomic stability are provided in Table 4. A possible conclusion from the *in vitro *assays is that when the zinc concentration used is below the human serum value (Table 2), the results tend to be beneficial for the cultured cells. In contrast, zinc concentrations above the physiological level can damage cultured cells. Again, it is interesting to observe that certain cell-culture media (e.g., HAM F-10 and F-12), even when supplemented with FBS, cannot provide enough of this micronutrient for appropriate cell development and genomic stability once the concentration falls below the physiological range.

## 6. What Must Be Done: Limitations of the Available Evidence and Conclusions

Micronutrients are clearly important for cell development and genomic stability, and many of the micronutrients mentioned are necessary for the DNA synthesis and repair mechanisms.  Table 5 provides an overview of the current data regarding the effects of deficiencies or excesses of the micronutrients addressed in this review on genomic stability. The micronutrient levels found in the discussed cell-culture media and the status of research on each micronutrient are also highlighted. Evidently, much research has been performed, but more specific studies focusing on cell cultures are still required.

Even though there are some highly enriched media available as basal media for serum-free cell culture, like Medium 199 or Ham F-12 nutrient mixture, the most common source of micronutrients currently used in cell cultures is still FBS. The limitations of FBS in providing adequate micronutrient concentrations have been analyzed and described in the literature [34]. Given that cell- and tissue-culture models are generally important in scientific research, the development of standards *in vitro* methods is mandatory. These new standards will decrease dependence on animal serum, a supplement with an undefined, variable composition that can considerably influence experimental results [209]. Furthermore, according to van der Valk et al. [209], an improved exchange of information regarding newly developed serum-free media may be beneficial. It has also become clear that nearly every cell type has distinct requirements for media supplementation, and especially, as discussed in this review, for micronutrient supplementation. A universal cell- and tissue-culture medium may not be feasible, as different cell types have different receptors involved in cell survival, growth and differentiation, and release different factors into the surrounding environment.

Besides this, it is important to highlight that although the formulations of the classical cell culture media are unchanged for a long time, since their development, the quality and purity of single components used as supplements, are likely to have increased considerably. However, some losses of important substances could have occurred, including trace elements, vitamins, growth factors, and lipids and this should be better addressed before defined a serum-free media. In fact, the threshold for developing and using a new well-defined medium, given that the current FBS-supplemented culture media work well, is high [209]. At the very least, an evaluation of FBS composition, in terms of micronutrients and possibly other factors, should be strongly considered in the laboratories that focus on *in vitro* studies. Knowledge of the micronutrient composition of FBS may help to minimize the bias in experimental results. However, maintaining both successful and consistent cell cultures can be difficult, as FBS is a complex natural product and may vary between batches, even if obtained from a single manufacturer. More specifically, the quality and concentration of both bulk and specific proteins in cell cultures can affect cell growth [210]. Adjusting the *in vitro* micronutrient levels to physiological values will guarantee a better environment for cell development, mimicking the *in vivo* milieu.

Further studies on the effects of micronutrients on cell viability, proliferation, and stability, as well as gene expression and integrity are still required, but the information already available is a sufficient call to action. As mentioned by Ferguson and Fenech [141], most investigations have been limited to studying the effects of single micronutrients and have not considered genetic consequences. Thus, there is an important need for studies that also examine nutrient-nutrient and nutrient-gene interactions. Determining the physiological range of such significant micronutrients as iron and then adjusting the concentrations currently found in cell-culture media may be beneficial for *in vitro *assays. More specifically, the viability and genomic stability of cell lines and primary cultures may be improved. Depending on the cell type (primary, immortalized, tumor, or normal) and origin (lung, hepatic, neural, or other), the requirement for a micronutrient may vary widely, so this subject should be carefully evaluated. Finally, the form of the micronutrient used in supplementation media may also influence experimental results. For example, according to Jacobs et al. [211], whether iron has toxic effects is directly related to the presence of a chelating agent, which reduces the concentration of free ferric ion and promotes the formation of ferritin.

Once the relationship between an *in vivo* imbalance of micronutrients and genomic stability, which may cause many diseases, including cancer, is established, it will be mandatory to better understand *in vitro* micronutrient supplementation. In fact, certain simple questions, such as *“is the concentration of this micronutrient sufficient for the development of this cell?”* or “*are the levels of this micronutrient similar to the levels observed in human serum?”,* may aid the proper design of *in vitro* studies.

## Figures and Tables

**Table 1:** Micronutrients linked to genomic stability, dietary requirements, and effects of deficiency and excess.

Micronutrient	EAR for adults (not pregnant or lactating)	General health effects of deficiency	Effects of deficiency related to genome instability	UL for Adults	Effects of excess related to genome instability	References
Vitamin						

Vitamin A	500–625 RAE	Blindness, impaired immunity, and dermal alterations	Increased sensitivity to DNA-damaging agents	3000 RAE	Congenital malformations while in pregnancy. Cancer risk increase for smokers	[48–50]
Vitamin B7 (biotin)	30 *μ*g*	Dermal alterations, immune dysfunction, neurological symptoms, and congenital malformations during pregnancy	Chromatin structural alterations	NA (safe up to 20,000 *μ*g)	Congenital malformations. Increase in DNA damage	[51–54]
Vitamin B9	320 DFE	Anemia and other hematological alterations, pregnancy complication (e.g., neural tube defect)	Uracil misincorporation in DNA; DNA strand breaks	1000 DFE	Increased cancer risk (promotion effect)	[43, 51, 55, 56]
Vitamin B12	2 *μ*g	From lack of energy to irreversible severe damage to nervous system	DNA strand breaks	1000 *μ*g	Unknown	[43, 51]
Vitamin C	60–75 mg (95–110 if smoker)	Dermatological alterations associated to collagen synthesis and immune impairment	DNA strand breaks	2000 mg	DNA damage related to oxidative stress	[43, 49, 55]
Vitamin E	12 mg	Increase in chronic disease risk	DNA strand breaks	1000 mg	DNA damage related to oxidative stress	[43, 49, 57, 58]

Mineral						

Copper	700 *μ*g	Anemia and other blood dysfunctions, impaired growth, and neurological alterations	Oxidative DNA damage increase	10000 *μ*g (under review)	DNA damage associated to oxidative stress, particularly to liver	[59, 60]
Iron	6–8.1 mg	Anemia and other blood dysfunctions, impaired growth, and neurological alterations	DNA damage increase	45 mg	DNA damage associated to oxidative stress, particularly to liver	[21, 43, 60]
Magnesium	255–350 mg	Rare because Mg deficiency is unusual	DNA repair deficiency	NA	Unknown	[61, 62]
Selenium	45 *μ*g	Decreased activity of glutathione peroxidase leading to increased risk of degenerative diseases and impairment in immunity	DNA strand breaks	400 *μ*g	Tumor incidence seems to be reduced in high doses supplementation	[49, 63]
Zinc	6.8–9.4 mg	Dermal alterations, growth retardation, immune dysfunction, neurological symptoms, night blindness, and adverse outcomes during pregnancy	DNA strand breaks	40 mg	DNA damage increase	[43, 60, 64]

*Adequate intake not EAR.

EAR: estimated adequate requirement; DFE: dietary folate equivalents; RAE: retinol activity equivalents; UL: upper level; NA: not available.

**Table 2:** Concentrations (in *μ*mol/L) of micronutrients that can increase genomic stability in traditional cell-culture media and FBS versus human serum.

Micronutrients	Cell culture medium*								10% FBS**	Mean human serum concentration***	Status cell-culture medium versus
	MEM	DMEM	L-15	M-199	HAM F-10	HAM F-12	RPMI-1640	DMEM/HAM F12			human serum
Vitamins											

Vitamin A	NA	NA	NA	3.1 × 10^−1^	NA	NA	NA	NA	3.0 × 10^−2^	2.0	Lower
Vitamin B7 (Biotin)	NA	NA	NA	4.1 × 10^−2^	1.0 × 10^−1^	3.0 × 10^−2^	8.2 × 10^−1^	1.0 × 10^−2^	Trace	4.0 × 10^−4^	Higher
Vitamin B9	2.3	9.1	2.3	2.3 × 10^−2^	3.0	3.0	2.3	6.0	Trace	5.0 × 10^−3^	Higher
Vitamin B12	NA	NA	NA	2.8 × 10^−1^	1.0	1.0	4.0 × 10^−3^	5.0 × 10^−1^	Trace	3.0 × 10^−4^	Higher
Vitamin C	NA	NA	NA	1.4 × 10^−2^	NA	NA	NA	NA	Trace	50.0	Lower
Vitamin E	NA	NA	NA	NA	NA	NA	NA	NA	0.0003	30.0	Unknown

Minerals											

Copper	NA	NA	NA	NA	1.0 × 10^−2^	1.0 × 10^−2^	NA	5.0 × 10^−3^	Trace	14.0	Lower
Iron	NA	2.5 × 10^−1^	NA	1.7	3.0	3.0	NA	1.6	3.0	23.0	Lower
Magnesium	8.0 × 10^2^	8.0 × 10^2^	1.8 × 10^2^	NA	6.2 × 10^2^	6.1 × 10^2^	4.1 × 10^2^	1.1 × 10^3^	Trace	8.0 × 10^2^	Lower/similar
Selenium	NA	NA	NA	NA	NA	NA	NA	3.0 × 10^−2^	3.0 × 10^−2^	11.0	Lower
Zinc	NA	NA	NA	NA	1.0 × 10^−1^	3.0	NA	1.5	Trace	17.0	Lower

NA: not available.

*MEM: minimum essential medium; DMEM: Dulbecco's modified eagle medium; L-15: Leibovitzs medium 15; M-199: medium-199; HAM F-10 and F-12: Ham's nutrient mixture F-10 and F-12; RPMI-1640: Roswell Park Memorial Institute medium; DMEM/HAM F-12: Dulbecco's modified eagle medium/Ham's nutrient mixture F-12. The vitamin and mineral concentrations described were obtained from the webpages of the key suppliers.

**The values for the vitamin A, vitamin E, and selenium concentration in FBS were found in [144], and the iron concentration in FBS was determined analytically.

***The references citing the micronutrient concentrations in human serum are as follows: vitamins A [145], B7 [146], B9 [147], B12 [148], C, and E [149]; Mg [150]; Cu, Fe, Se, and Zn [151]. The concentration of the vitamins and minerals in the media were obtained from the manufacturers.

**Table 3:** Examples from the literature of vitamins' effects in cell culture and on genomic stability.

Micronutrient	Main effects on cell viability and genomic stability	Cell type	Additional information regarding the form and concentration of the micronutrient evaluated	Status in relation to physiological concentration	References
Vitamin A	Enhanced the levels of 8-oxo-dG DNA damage but significantly inhibited M1dG formation especially after induction of M1dG by H_2_O_2_ or B[a]P; increased production of reactive oxygen species and formation of promutagenic DNA lesions	Lung epithelial cells	Beta-carotene (5 μmol/L)	Similar	[159, 160]
	Caused oxidation of dG and cytotoxicity, giving rise to an almost complete cell death	Leukemia cells (HL-60)	Retinol (2 μmol/L) and ascorbic acid (50 μmol/L)	Similar	[161]
	Induced apoptosis by increasing apoptotic protein p53 and decreasing antiapoptotic Bcl-2 as well as nuclear ATM; also induced DNA fragmentation	Gastric cancer cells (AGS)	Beta-carotene (100 μmol/L)	Higher	[162]
	DNA damage on HepG2 which was also concordant to increased apoptosis and necrosis of cells	Hepatocarcinoma cells (HepG2)	Beta-carotene (4 μmol/L) and 8 μmol/L)	Similar	[163, 164]
	Reduced levels of total DNA adducts and increased apoptosis levels in cells coexposed to benzo(a)pyrene and retinoic acid		Retinoic acid (1 μmol/L)	Lower	

Vitamin B7 (biotin)	Increased strand breaks and cellular response to strand breaks	T-lymphocyte cell line (Jurkat)	25 × 10^−6^ μmol/L and 0.01 μmol/L	Lower and higher	[75]
	Affects biotinylation of proteins, gene expression, and metabolism of interleukin-2; rates of proliferation and apoptosis were not affected by biotin status		25 × 10^−6^ μmol/L, 25 × 10^−5^ μmol/L and 0.01 μmol/L	Lower and higher	[165]

Vitamin B9	Increased levels of excision repair and apoptosis	Lymphocytes	Folate (<2.3 × 10^−3^ μmol/L)	Lower	[166, 167]
	Decreased apoptosis and increased cell proliferation	Neural stem cells (NSCs)	Folic acid (8.4 × 10^3^ μmol/L)	Higher	[168, 169]
	High concentration accelerated growth; increased metabolic activity, proliferation, and apoptosis; decreased differentiation	Human colon cancer cells (HT29)	Folic acid (0.021 μmol/L and 0.21 μmol/L) with other micronutrients involved in folate-methionine cycle	Similar and higher	[56]

Vitamin B12	Reduced cell proliferation and increased differentiation	Neuroblastoma cells (NIE115)	Vitamin B12 (total absence)	Lower	[89]
	Chronic exposure inhibited neurotoxicity	Retina cells (primary cultures from fetal rats)	Methylcobalamin (1 μmol/L)	Higher	[170]
	Absence is likely to result both in reduced cell proliferation and in cell death, as inhibition of DNA synthesis generally results in apoptosis	Human erythroleukemic (K562) and murine lymphoma (BW5147) cell lines	Cobalamin (total absence and 3.7 × 10^−3^ μmol/L)	Lower and higher	[171]

	Physiological concentrations of AA were not toxic, while high concentrations of AA induced DNA strand breakage in a dose-dependent manner, whereas AA2P were not genotoxic	Human dermal fibroblasts (HDFs)	Ascorbic acid (AA) and ascorbic acid 2-Phosphate (AA2P) (total absence or 20, 100, and 500 μmol/L)	Lower, similar, and higher	[172]
Vitamin C	Enhanced DNA-protein crosslinks and cytotoxicity	Chinese hamster cells (V79)	Ascorbic acid (1000 μmol/L)	Higher	[173]
	Decreased number of 8-hydroxydeoxyguanosine adducts	Mouse keratinocyte cell line	Ascorbic acid (2,27 μmol/L and 4,54 μmol/L)	Lower	[174]
	Protective effect against DNA damage induced by X-ray treatment	Human lymphoblastoid cells (Raji)	Ascorbic acid (60 μmol/L)	Similar	[175]

	Protective effect against DNA damage induced by H_2_O_2_ treatment	Raji cells	α-Tocopherol (30 μmol/L)	Similar	[175]
Vitamin E	Reduced DNA fragmentation and apoptotic body formation, possibly favoring DNA repair	African green monkey kidney (Vero), human colon carcinoma (Caco-2), and dysplastic oral keratinocyte (DOK) cells	Vitamin E (25 μmol/L)	Similar	[176]
	Reduced apoptosis and autophagy	Cultured trophoblasts and villous explants obtained from human placentas at term	Vitamin E (50 μmol/L) with vitamin C (50 μmol/L)	Higher	[177]

**Table 4:** Examples from the literature of minerals' effects in cell culture and on genomic stability.

Micronutrient	Main effects on cell viability and genomic stability	Cell type	Additional information regarding the form and concentration of the micronutrient evaluated	Status in relation to physiological concentration	References
	Increased cytotoxicity and ROS formation	HepG2	50, 100, 150, and 200 μmol/L	Higher	[184]
Copper	Reduced mitochondrial activity and cell viability and increased DNA damage	Chinese hamster ovary cells (CHO-K1)	24.55, 35.40, 48.31, 89.23, 116.77, 170.75, 339.45, and 450.35 μmol/L	Higher	[185]
	Increased the DNA damage in a dose-dependent manner and also reduced rates of DNA synthesis and histone acetylation	Leukemia cells (HL-60)	Total absence, 10, 20, 50, 100 and 200 μmol/L	Lower, Similar, and Higher	[186]

	Inhibited DNA synthesis in proliferative cells	Human lymphocytes	Iron suiphate (22.38, 44.76, and 89.52 μmol/L)	Similar and Higher	[187]
Iron	Possibly accelerated aging process and death at concentrations >10 *μ*mol/L, whereas 5 *μ*mol/L increased protein content	Cerebellar granule cells	Ferric nitrilotriacetate (5, 10, 15, 20, and 40 μmol/L are shown	Lower, Similar, and Higher	[188]
	Genotoxic effects	Primary nontransformed colon cells and preneoplastic colon adenoma cell line (LT97)	Ferric nitrilotriacetate (10, 100, 250, 500, and 1000 μmol/L)	Lower and Higher	[189]

	Inhibited cell proliferation and promoted endothelial dysfunction by generating proinflammatory, prothrombotic, and proatherogenic environment	Human endothelial cells	Magnesium sulphate (100, 500, and 1000 μmol/L)	Lower and Higher	[190]
	Inhibited growth more drastically in normal than in transformed cells and altered cell-cycle progression	Normal (HC11) and transformed (MCF-7) breast epithelial cell lines	Total absence, 10, 30, 50, 100, 300, and 500 μmol/L	Lower	[191]
Magnesium	Inadequate concentration accelerated cell senescence	Normal human fibroblasts (IMR-90)	100, 400, and 800 μmol/L	Lower and Similar	[192]
	Incision repair completely inhibited in absence of Mg^2+^ as well as at very high concentrations, whereas optimal concentrations essential in all steps of NER	Human lymphoblastoid (AHH1) and clonal human epithelial adenocarcinoma (HeLa S3) cell lines	400 and 800 μmol/L	Lower and Similar	[193]

Selenium	Methylseleninic acid, L-selenocysteine, selenodiglutathione, or selenite-induced cell death in micromolar concentrations, whereas selenomethionine or ebselen was not toxic within the concentration range tested	HepG2, human hepatoma cell line (Huh-7), and mouse hepatoma (Hepa 1-6)	Sodium selenite, L- or DL-selenocysteine, selenodiglutathione, selenomethyl-selenocysteine, sodium selenate, L- or DL-selenomethionine, methylseleninic acid, ebselen, selenomethionine, and selenodiglutathione (0.1 × 10^−3^ to 1000 μmol/L)	Lower, Similar and Higher	[194]
	Induces G1-cell cycle arrest and apoptosis via multiple signaling pathways, which may play a key role in methylselenol-induced inhibition of cancer cell proliferation and tumor cell invasion	Human sarcoma cell line (HT1080)	Seleno-L-methionine (SeMet) (total absence, 1.25, 2.5, and 5 μmol/L)	Lower	[195]
	Decrease in cell damage and protection against oxidative stress	HepG2 cells	Selenium methylselenocysteine (0.01, 0.1, 1, and 10 μmol/L)	Lower and Similar	[196]
			Selenium methylselenocysteine (1 μmol/L)	Lower	[197]

	Increased oxidative DNA damage; disrupted p53, NF*κ*B, and AP1 DNA binding; decreased DNA repair	Rat glioma cell line (C-6)	Zn sulfate and Zn carnosine (4.0 μmol/L)	Lower	[198]
Zinc	Decreased cell growth and viability, increased DNA SB and cytotoxicity in Zn-depleted cultures as well as at concentrations of 32 and 100 *μ*M; reduced genomic damage in cultures supplemented with 4 or 16 *μ*M	Human lymphoblastoid cell line (WIL2-NS)	Zn sulfate and Zn carnosine (total absence, 0.4, 4.0, 16.0, 32.0, and 100.0 μmol/L)	Lower, Similar, and Higher	[199]
	Decreased cell viability in Zn-depleted cultures (0 *μ*M) as well as at concentrations of 32 and 100 *μ*M for both Zn compounds and increased DNA SB, apoptotic, and necrotic cells in Zn-depleted cultures	Primary human oral keratinocyte cell line (HOK)			[200]

**Table 5:** Overview of the data addressed in this review.

Micronutrient	Evidence of genomic instability induction		Concentration in common cell-culture media versus physiological concentration	Optimal concentration proposed for cell culture
	Deficiency	Excess		
Vitamin A	+	+	Lower	Studied
Vitamin B7	+	+	Higher	Requires more studies
Vitamin B9	+	+	Higher	Studied
Vitamin B12	+	NA	Higher	Studied
Vitamin C	+	+	Unknown	Studied
Vitamin E	−	+	Unknown	Studied
Copper	+	+	Lower	Studied
Iron	+	+	Lower	Studied
Magnesium	NA	+	Similar	Studied
Selenium	+	−	Lower	Studied
Zinc	+	+	Lower	Studied

NA: Not available.

(−) Negative: the available data indicate no effect.

(+) Positive: the available data indicate an effect.
